# Oxidation Behavior of Pre-Strained Polycrystalline Ni_3_Al-Based Superalloy

**DOI:** 10.3390/ma17071561

**Published:** 2024-03-29

**Authors:** Rui Guo, Jian Ding, Yujiang Wang, Haomin Feng, Linjun Chen, Jie Yang, Xingchuan Xia, Yingli Zhao, Jun Li, Shuang Ji, Junyi Luo

**Affiliations:** 1 School of Material Science and Engineering, Hebei University of Technology, Tianjin 300130, China; guorui0404@163.com (R.G.); fhmwork@163.com (H.F.); yangjie07272023@163.com (J.Y.); 2 National Key Laboratory for Remanufacturing, Army Academy of Armored Forces, Beijing 100072, China; hitwyj@126.com; 3 Advanced Technology & Materials Co., Ltd., Beijing 101318, China; chenlinjun@atmcn.com (L.C.); junli@atmcn.com (J.L.); luojunyi@atmcn.com (J.L.); 4 HBIS Group Co., Ltd., Shijiazhuang 050023, China; zhaoyingli@hbisco.com (Y.Z.); jishuang@hbisco.com (S.J.)

**Keywords:** Ni_3_Al-based superalloys, pre-strain, microstructure, dislocation, antioxidant properties

## Abstract

The harsh service environment of aeroengine hot-end components requires superalloys possessing excellent antioxidant properties. This study investigated the effect of pre-strain on the oxidation behavior of polycrystalline Ni_3_Al-based superalloys. The growth behaviors of oxidation products were analyzed by scanning electron microscope, transmission electron microscope, X-ray Photoelectron Spectroscopy and Raman spectroscopy. The results indicated that the 5% pre-strained alloys exhibited lower mass gain, shallower oxidation depth and more compact oxide film structures compared to the original alloy. This is mainly attributed to the formation of rapid diffusion paths for Al atoms diffusing to the surface under 5% pre-strain, which promotes the faster formation of protective Al_2_O_3_ film while continuing to increase the pre-strain to 25% results in less protective transient oxidation behavior being aggravated due to the increase in dislocation density within the alloy, which prevents the timely formation of the protective Al_2_O_3_ film, resulting in uneven oxidation behavior on the alloy.

## 1. Introduction

With the increasing thrust-weight ratio of advanced aeroengines, the operating temperature of the combustion chamber also rises, leading to higher demands for high-temperature structural materials [1,2,3]. Ni-based superalloys are widely used for high-temperature structural components in aeroengines, constituting approximately 40~50% of the usage [4]. However, the service temperature of traditional Ni-based superalloys is already close to its limitation, reaching 85~90% of their melting points, and further improvements in service temperature are limited [5]. NiAl and Ni_3_Al are promising intermetallic compounds according to the Ni-Al binary system, due to their higher melting points (1395 °C and 1639 °C, respectively) [6,7]. Polycrystalline Ni_3_Al-based superalloys, mainly composed of the intermetallic compounds NiAl (β phase, B2-type structure) and Ni_3_Al (γ′ phase, L1_2_-tybe structure), have gained significant attention in the field of high-temperature structural materials [8]. These alloys offer advantages such as lower density, high melting point and excellent high-temperature performance, making them potential replacements for traditional heat-resistant materials [9,10,11]. Researchers have conducted studies on the microstructure of polycrystalline Ni_3_Al-based superalloys. It has been proved that annealing treatment can promote precipitation of quasi-spherical α-Cr phase particles within the β phase, resulting in improved creep properties [9]. In addition, the morphology of the reinforcing phase, known as the γ′ phase, in polycrystalline Ni_3_Al-based superalloys also significantly influences its properties. He et al. [12] investigated the formation of various morphologies, including spherical, cubic and concave–cubic shapes of initial the γ′ phase. Furthermore, Ding et al. [13] reported that the formation of mushroom-like γ′ phases in the γ′-envelope region during cyclic heat treatment is mainly attributed to vacancies, small-angle grain boundaries, dislocations and segregation of aluminum elements. Researchers have conducted studies on the deformation behavior of Ni_3_Al-based superalloys. Kobayashi et al. [14] examined the local deformation characteristics of 95% cold-rolled Ni_3_Al foils using tensile and bending tests. The peak strain value at the initiation of the crack in both tests was 10%. The bending test demonstrated higher ductility compared to the tensile test. Additionally, Polkowski et al. [15] reported that Ni_3_Al-based alloys exhibit increased mechanical strength after undergoing a process of differential speed rolling compared to the same material deformed through other methods.

Aeroengine hot-end components undergo alloy oxidation during servicing at high temperatures, which can cause substantial damage and failure [16,17]. As a result, researchers have focused on enhancing the antioxidant properties of superalloys. Due to varying thermodynamic and kinetic conditions of different oxides, distinct oxidation forms will manifest on the surface. For instance, the outer NiO and inner Al_2_O_3_ structures formed during the oxidation process of Ni_3_Al-based superalloys play a crucial role in determining the alloy’s antioxidant properties [18]. In addition to applying protective layers to the matrix [19,20,21], the antioxidant properties of the alloys can be enhanced through the processes of heat treatment [8,22,23] and alloying [24,25]. These methods have achieved good results, although it is challenging to achieve further significant improvements in the antioxidant properties of superalloys. Recently, it was reported that pre-strain can introduce defects such as interfaces and dislocations within the alloys [26] and these defects possess high energy levels which can facilitate the diffusion of elements and preferential formation of oxidation products [27].

Generally, the excellent antioxidant properties for most superalloys are mainly attributed to the formation of protective oxide films [10]. By controlling the formation of protective oxide film through the pre-strain process, it is possible to enhance the antioxidant properties of alloys. Mayank et al. [28] demonstrated that the antioxidant properties of high entropy alloys were significantly improved through surface strain. However, the effect of overall strain on the antioxidant properties is not well understood compared to surface strain. As far as we know, there are two different perspectives on the effect of pre-strain. One perspective thinks that the oxidative damage level is increased by pre-strain, leading to cracking and spalling in scales [29,30,31]. Other researchers have presented a different perspective. Wang et al. [32] proved that pre-strain promoted the growth of stable protective oxide film on a high entropy alloy, resulting in improved antioxidant properties. Qi et al. [33] observed that a DZ125 alloy subjected to tensile pre-strain exhibited thinner oxide layers and less weight gain. Similarly, Barnard et al. [34] found that HAYNES 75 and HAYNES 230 alloys exhibited lower mass change and thinner oxide layers when pre-strain was applied. As described above, pre-strain may offer a promising approach to enhance the antioxidant properties of alloys. It is crucial to further investigate its real effect. Nevertheless, up to now, the impact of pre-strain on the oxidation behavior of polycrystalline Ni_3_Al-based superalloys has not yet been established and further research is needed.

In this work, the oxidation behavior of polycrystalline Ni_3_Al-based superalloys after pre-strain through compression testing at 1100 °C was investigated. Meanwhile, we present a systematic analysis of the oxidation behavior of pre-strained alloys in each phase region during short-term oxidation and the effect of dislocations generated by pre-strain on oxidation behavior. Furthermore, we combine oxidation kinetics and the morphology of oxidation products to analyze the evolutionary behavior of oxidation products formation in pre-strained alloys during the long-term cyclic oxidation process. The study reveals the oxidation mechanism of pre-strained alloys and discusses the factors contributing to oxidation resistance.

## 2. Materials and Methods

### 2.1. Material and Specimen Preparation

The polycrystalline Ni_3_Al-based superalloy used in this work was developed by adjusting the content of W, Mo, Ti and Hf elements on the basis of an IC396 alloy, and the chemical composition is shown in Table 1. Vacuum induction melting (VIM) and electroslag remelting (ESR) were used to prepare the ingot. After that, the ingot was homogenized at 850 °C for 2 h (hereinafter referred to as the original alloy).

Compression testing was used for pre-strain. A WDW-200 universal testing machine was used according to GB/T 7314-2017 [35]. To ensure consistency, all samples were cut into cylinders with 8 mm diameter and 12 mm height using wire electrical discharge machining (WEDM) from the ingot possessing similar solidification conditions. Displacement control was utilized during the compression process with a compression rate of 0.72 mm/min, resulting in predetermined deformation of 5% and 25%. The corresponding pressure values were 1.82 kN and 68.82 kN, respectively. Samples were cut longitudinally along the compression direction, ground, polished and then etched with Nimonic reagent (12.5 g CuCl_2_ + 12.5 g FeCl_2_ + 50 mL HNO_3_ + 200 mL HCl) for 3–5 s. Afterward, specimens were washed with distilled water and alcohol, and dried with cold flowing air. Subsequently, the longitudinally cut specimens were polished until the surface was free of scratches and then exposed to a high-temperature oxidation environment.

### 2.2. Oxidation Test

All the oxidation tests were conducted using a high-temperature box-type resistance furnace with a maximum operating temperature of 1300 ± 1 °C. It is important to note that the furnace was preheated to 1100 °C for over 20 h to ensure temperature stability. For short-term oxidation, both the original and pre-strained alloys were subjected to oxidation at 1100 °C for 5 min and 30 min. Prior to long-term cyclic oxidation experiments, the initial weights of the original and pre-strained specimens were determined using an electronic scale with the accuracy of 0.0001 g. Subsequently, the specimens were placed in a box-type resistance furnace set at 1100 °C. All the specimens were taken out every 10 h, air-cooled to room temperature and weighed. A total duration of 200 h was used for long-term cyclic oxidation. 

### 2.3. Microstructure Characterization

Microstructure observation was conducted using a 7610F scanning electron microscope (SEM, JEOL Company, Beijing, China) and an OLYCIA M3 metallographic microscope (OM, Beijing Daqi Zongheng Tech. Co. Ltd., Beijing, China). The oxidation microstructure and oxidation products were analyzed using a JSM-6510A lanthanum hexaboride scanning electron microscope (SEM, JEOL Company, Beijing, China) and energy dispersive spectrometer (EDS, JEOL Company, Beijing, China). The microstructure and dislocation state were observed using a JEM 2100F transmission electron microscope (TEM, JEOL Company, Beijing, China). The specimens for TEM observation were initially cut into a thin sheet with a thickness of approximately 300 μm using wire electrical discharge machining and then mechanically ground to ~50 μm, followed by punching into discs with a 3 mm diameter using a sample puncher. Subsequently, an electrolytic double jet thinning instrument (with voltage of 40 V) was used to thin specimens with a double injection liquid consisting of 10 vol % HClO_4_ + 90 vol % C_2_H_5_OH at −30 °C. The internal defects and oxidation products were analyzed using a Bruker D8 Advance X-ray diffractometer (XRD, Rigaku Corporation, Beijing, China) with Cu-Kα radiation, employing a scanning speed of 6°/min. The composition of oxidation products within a displacement range of 100–1000 cm^−1^ was analyzed using LabRAM HR Evolution laser Raman spectroscopy (HORIBA, Shanghai, China) with an excitation wavelength of 532 nm and laser power of 50 mW.

## 3. Results

### 3.1. Microstructure of Original Alloy

**Figure 1:**
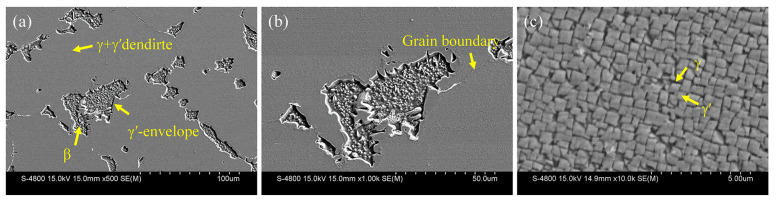
Microstructure of the original alloy: (**a**) overall morphology; (**b**) β phase and γ′-envelope; (**c**) (γ + γ’) dendrite.

Figure 1 shows the microstructure of the original alloy; it can be seen that the alloy primarily consists of three regions: the interdendritic β phase region, (γ + γ′) dendritic region and γ′-envelope region (Figure 1a). The β phase exhibits an island-like morphology and is surrounded by a γ′-envelope (Figure 1b). Meanwhile, numerous uniform cubic γ′ phases uniformly distribute within (γ + γ′) dendrites (Figure 1c). These γ′ phases are interconnected through γ phases, also known as γ channels (Figure 1c).

### 3.2. Microstructure of Short-Term Oxidation Products

**Figure 2:**
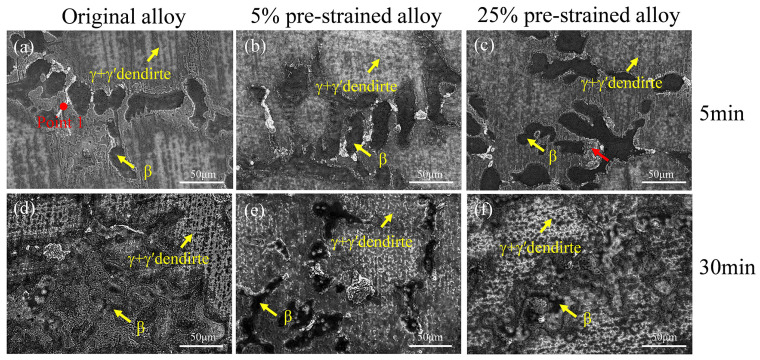
Oxidation morphologies of the original and pre-strained alloys oxidized at 1100 °C for 5 min and 30 min: (**a**) original alloy_5 min; (**b**) 5% pre-strained alloy_5 min; (**c**) 25% pre-strained alloy_5 min; (**d**) original alloy_30 min; (**e**) 5% pre-strained alloy_30 min; (**f**) 25% pre-strained alloy_30 min. The red arrow shows oxidation aggregation.

Figure 2 shows the surface morphologies of the original and pre-strained alloys after oxidation at 1100 °C for 5 min and 30 min, respectively. It is clear that different regions exhibit distinct oxidation morphologies. After 5 min of oxidation, the boundaries of each phase region are relatively distinct for the original alloy (Figure 2a). The β phase undergoes slight oxidation, while oxidation mainly occurs in the γ’-envelope between the β phase and (γ + γ’) dendrite. Additionally, a small amount of white oxide appears near this area, as indicated by point 1 in Figure 2a. In comparison, the oxidation products noticeably decrease in the γ’-envelope region in the 5% pre-strained alloy (Figure 2b). For the alloy with pre-strain of 25%, oxidation products in the γ’-envelope increase, with some areas showing aggregation, as indicated by the red arrow in Figure 2c. After 30 min of oxidation, the oxidation products of the original alloy expand and grow, making it challenging to distinguish the boundaries of each phase region (Figure 2d), while for the alloy with pre-strain of 5%, the boundaries of each phase region can still be distinguished (Figure 2e), indicating that pre-strain inhibits the growth of oxidation products. On the other hand, when the pre-strain reaches 25%, although the oxidation products in γ’-envelope region decrease, rapid growth of oxidation products in the β phase makes it difficult to distinguish the boundaries of each phase region (Figure 2f).

**Figure 3:**
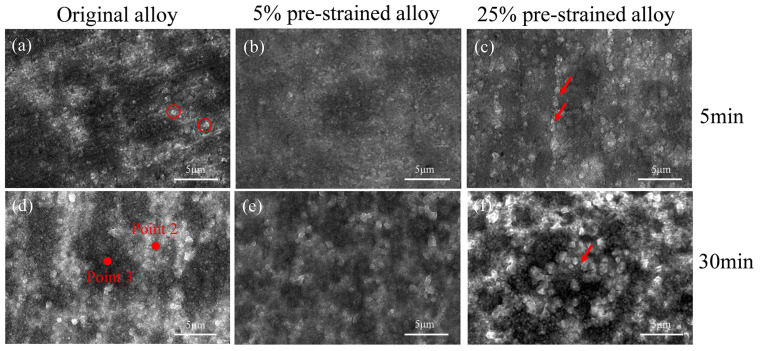
Oxidation morphologies in (γ + γ′) dendritic region of the original and pre-strain alloys after oxidation at 1100 °C for 5 min and 30 min: (**a**) original alloy_5 min; (**b**) 5% pre-strained alloy_5 min; (**c**) 25% pre-strained alloy_5 min; (**d**) original alloy_30 min; (**e**) 5% pre-strained alloy_30 min; (**f**) 25% pre-strained alloy_30 min. The red circles are oxidized particles and the red arrow shows oxidation aggregation.

Figure 3 illustrates the oxidized morphology of the (γ + γ′) dendritic region in both the original and pre-strained alloys after 5 min and 30 min of oxidation at 1100 °C. After 5 min of oxidation, small granular oxides with some aggregation appear in the (γ + γ′) dendritic region of the original alloy, as indicated by the red circle in Figure 3a. The oxidized aggregation weakens in the 5% pre-strained alloy, while the dimension of granular oxides decreases and is more uniformly distributed (Figure 3b). For the alloy with pre-strain of 25%, the dimension of granular oxides increases compared to that of the original alloy, and the aggregation reappears, as indicated by the red arrow in Figure 3c. After 30 min of oxidation, the granular oxides in the original alloy continue to aggregate and grow, and more noticeable oxidation bumps appear (Figure 3d). For the alloy with pre-strain of 5%, granular oxides distribute more uniformly compared to the original alloy, although their dimensions increase compared to those after 5 min of oxidation (Figure 3e). For the alloy with pre-strain of 25%, the dimension of granular oxides increases and the oxidized aggregation phenomenon becomes more pronounced compared to that after 5 min of oxidation, as indicated by the red arrow in Figure 3f.

**Figure 4:**
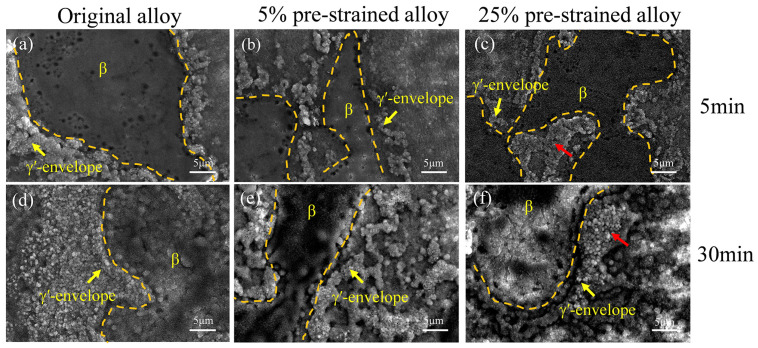
Oxidation morphologies in β phase and γ′-envelope region of the original and pre-strained alloys after oxidation at 1100 °C for 5 min and 30 min: (**a**) original alloy_5 min; (**b**) 5% pre-strained alloy_5 min; (**c**) 25% pre-strained alloy_5 min; (**d**) original alloy_30 min; (**e**) 5% pre-strained alloy_30 min; (**f**) 25% pre-strained alloy_30 min. The red arrow shows oxidation aggregation.

Figure 4 illustrates the oxidized morphologies of the β phase and γ′-envelope regions in both the original and pre-strained alloys at 1100 °C for 5 min and 30 min. After 5 min of oxidation, there are fewer oxidation products in the β phase due to the short oxidation time. However, the oxidation is more pronounced for the original alloy’s γ′-envelope region, even leading to aggregation. As the pre-strain increases, oxidation products in the γ′-envelope region decrease significantly (Figure 4a–c). The most significant reduction appears in the alloy with a pre-strain of 5%. Oxidized products in certain regions aggregate in the alloy with pre-strain of 25%, as indicated by the red arrow in Figure 4c. After 30 min of oxidation, the oxide film in the β phase region of the original alloy exhibits holes (Figure 4d). Conversely, the oxide film in the β phase region of the 5% pre-strained alloy is uniform and dense (Figure 4e). The oxide film in the β phase region of the 25% pre-strained alloy becomes cracked (Figure 4f). Furthermore, the oxidation of the γ′-envelope region of the original alloy intensifies with longer oxidation times, causing it to expand and grow towards the (γ + γ′) dendritic regions. In comparison to the original alloy, oxidation products in the 5% pre-strained alloy reduce in the γ′-envelope region and present stripe patterns. When the pre-strain reaches 25%, the oxidation products in γ′-envelope region reduce, but aggregation growth appears, as indicated by the red arrows in Figure 4f.

### 3.3. Oxidation Kinetics of Long-Term Cyclic Oxidation

**Figure 5:**
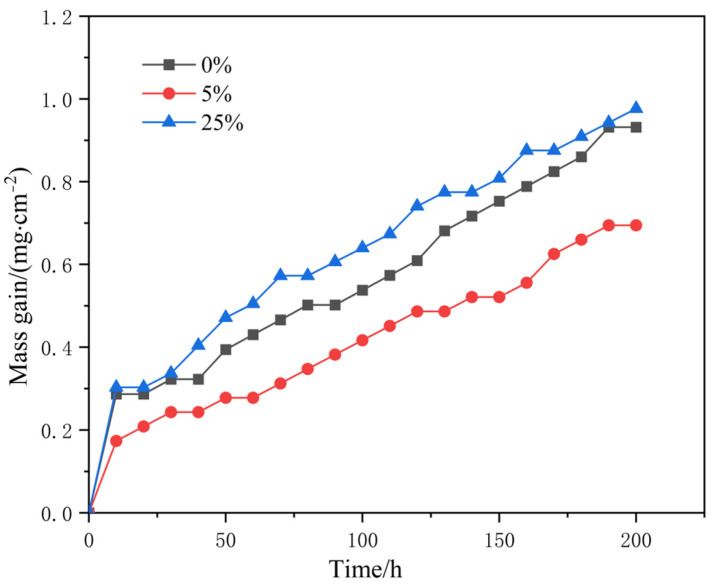
Oxidation kinetics of the original and pre-strained alloys at 1100 °C.

Figure 5 illustrates the weight gains of the original and pre-strained alloys after long-term cyclic oxidation. The results show that the weight gains are 0.9319 g/cm^2^, 0.6944 g/cm^2^ and 0.9764 g/cm^2^ for the original alloy, 5% and 25% pre-strained alloys, respectively. Notably, the weight gains of the 5% pre-strained alloy were significantly lower than those of the original alloy and 25% pre-strained alloy, which is consistent with the surface morphology observations in Figure 3, Figure 4 and Figure 5, indicating that 5% pre-strain enhances the antioxidation properties of the alloy.

## 4. Discussion

### 4.1. Oxidation Kinetics of Long-Term Cyclic Oxidation

**Figure 6:**
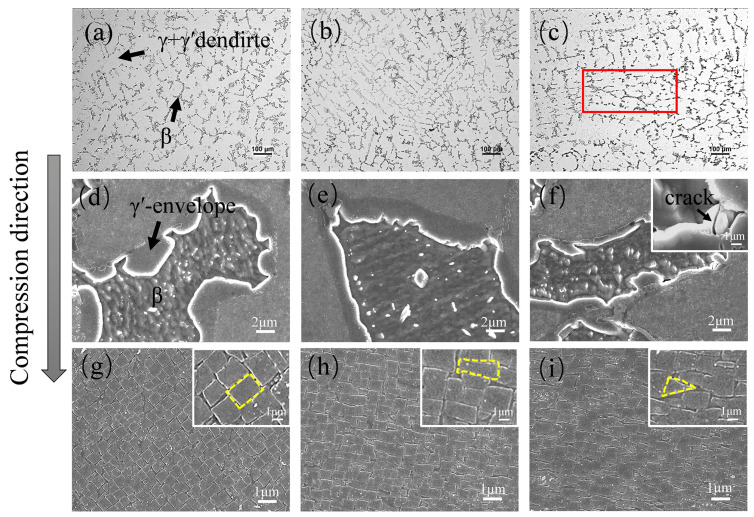
Effect of pre-strain on the microstructure of polycrystalline Ni_3_Al-based alloys: (**a**,**d**,**g**) original alloy; (**b**,**e**,**h**) 5% pre-strained alloy; (**c**,**f**,**i**) 25% pre-strained alloy. The red boxes show the region of β phase deformation.

Figure 6 illustrates the microstructure evolution of the original and pre-strained alloys. It is evident that the morphology of the β phase undergoes noticeable changes (Figure 6a–c). As the pre-strain increases, the β phase becomes smaller. Meanwhile, when the pre-strain reaches 25%, the β phase exhibits tensile deformation perpendicular to the compression direction, as indicated by the red box in Figure 6c. The difference between the β phase and γ’-envelope creates distinct interfaces which promote the diffusion of oxygen and metal ions due to higher energy at the interface [36], resulting in more pronounced oxidation in the γ’-envelope region, as illustrated in Figure 4. In addition, the γ′-envelope displays a folded morphology towards the β phase side (Figure 6d). The folded morphology becomes sharper, with pre-strain increasing (Figure 6d–f) and cracks appearing when the pre-strain is 25% (Figure 6f). This indicates that 25% pre-strain destroys the structural stability of the alloy, and it is not appropriate to increase the pre-strain. The presence of cracks will lead to the accumulation of oxidation products, which has a negative impact on the antioxidant properties of the alloy [27,30], leading to oxidation aggregation in the γ′-envelope region of the 25% pre-strained alloy (Figure 4f). Additionally, the (γ + γ′) dendritic region mainly consists of a cubic γ′ phase and γ channel between the γ′ phases (Figure 1c). For the alloy with 5% pre-strain, the γ′ phase undergoes deformation, transforms from cubic to approximate strip shape and becomes more compact. The γ channel also becomes narrower which facilitates the formation of protective oxide film [37], which is beneficial for the inhibition of oxidation product growth in the (γ + γ′) dendrite region, as shown in Figure 3b. For the alloy with 25% pre-strain, the γ′ phase transforms from a cubic to triangular shape. This is attributed to the increased lattice mismatch between γ and γ′ due to the pre-strain, resulting in short-range internal stresses within the crystal lattice and the stress causes the deformation of the γ′ phase under uniaxial external loading [38]. In addition, elemental diffusion is accelerated in regions with high lattice mismatch [12,39], leading to the formation of granular oxide aggregates (as shown in Figure 3f).

### 4.2. Growth Behavior of Short-Term Oxidation Products

**Figure 7:**
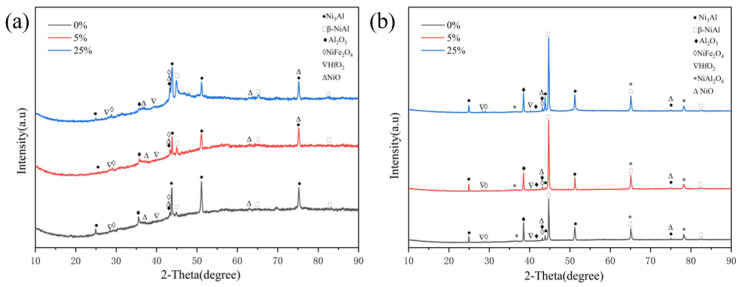
Phase composition of the original and pre-strained alloys after oxidation at 1100 °C: (**a**) 5 min; (**b**) 30 min.

Figure 7 shows phase compositions of the original and pre-strained alloys after oxidation at 1100 °C for 5 min and 30 min, respectively. It can be seen that the pre-strain process has no significant effect on the type of oxidation product. After 5 min of oxidation, the oxidation products on both the original and pre-strained alloys are Al_2_O_3_, NiO, NiFe_2_O_4_ and HfO_2_, while after 30 min of oxidation, unstable spinel NiAl_2_O_4_ appears due to the reaction between Al_2_O_3_ and NiO [22].

**Figure 8:**
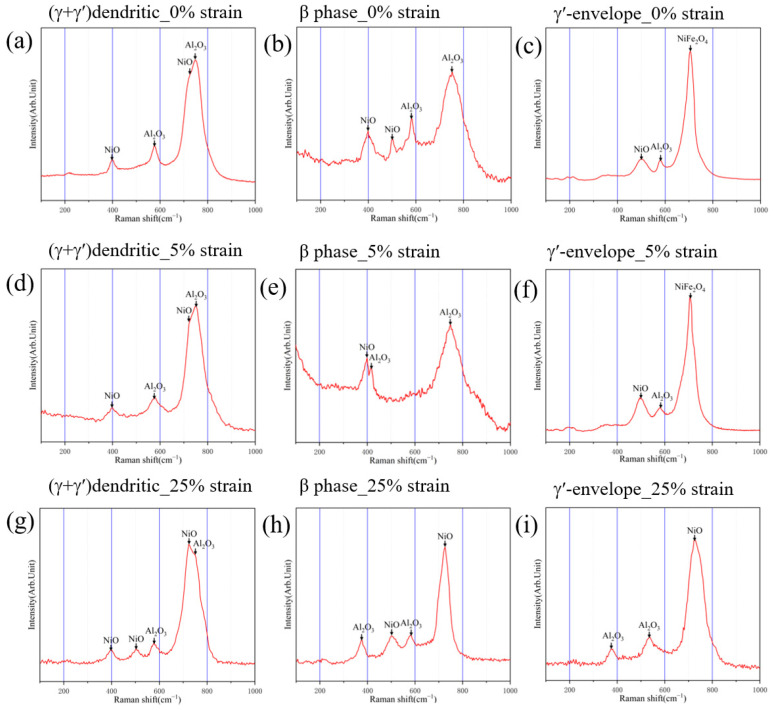
Oxidation products in different regions after oxidation at 1100 °C for 30 min.

As mentioned in Section 3.2, pre-strain affects the oxidation morphologies of different regions. To further investigate its impact, Raman spectroscopy was conducted on different regions of the original and pre-strained alloys, as illustrated in Figure 8. Figure 8a,d,g demonstrate the oxidation products in the (γ + γ′) dendritic region of the original and pre-strained alloys. It can be seen that four peaks appear at 400 cm^−1^, 578 cm^−1^, 725 cm^−1^ and 750 cm^−1^ for the original and 5% pre-strained alloys which correspond to NiO and Al_2_O_3_, with NiO peaks located at 400 cm^−1^ and 725 cm^−1^ [40,41] and Al_2_O_3_ peaks located at 578 cm^−1^ and 750 cm^−1^ [42,43], indicating that the oxidation products in the (γ + γ′) dendrite region are mainly NiO and Al_2_O_3_. Combined with the EDS analyses of point 2 and point 3 in Figure 3d (Table 2), it can be inferred that the granular oxides in the (γ + γ′) dendrite region are NiO, which has been proved by other researchers [10]. It should be noted that a characteristic peak at 500 cm^−1^ appears for the 25% pre-strained alloy, corresponding to NiO [44]. Compared with the original and 5% pre-strained alloy, the NiO peak for the 25% pre-strained alloy in the (γ + γ′) dendritic region is stronger than the peak of Al_2_O_3_. Generally, the peak intensity represents the content to some extent, indicating that the 25% pre-strained alloy has a higher content of NiO, which is also supported by its morphology (Figure 3f). The oxidation products in the β phase region of the original and pre-strained alloys are mainly NiO and Al_2_O_3_, as shown in Figure 8b,e,h. The most prominent peaks observed in the original and 5% pre-strained alloys are Al_2_O_3_ peaks, indicating a higher content of Al_2_O_3_ due to the higher Al content in the β phase, while for the alloy with pre-strain of 25%, the most prominent peak is a NiO peak, indicating that more NiO appears. Figure 8c,f,i demonstrate the oxidation products of the original and pre-strained alloys in the γ’-envelope region, which mainly consist of NiFe_2_O_4_, NiO and Al_2_O_3_. However, no characteristic peaks of NiFe_2_O_4_ are found in the 25% pre-strained alloy, indicating that pre-strain inhibits the formation of NiFe_2_O_4_. Furthermore, based on the XRD and EDS analysis (Table 2), it can be concluded that the white oxides observed in Figure 2 are HfO_2_ which has been proved by other researchers [45].

**Figure 9:**
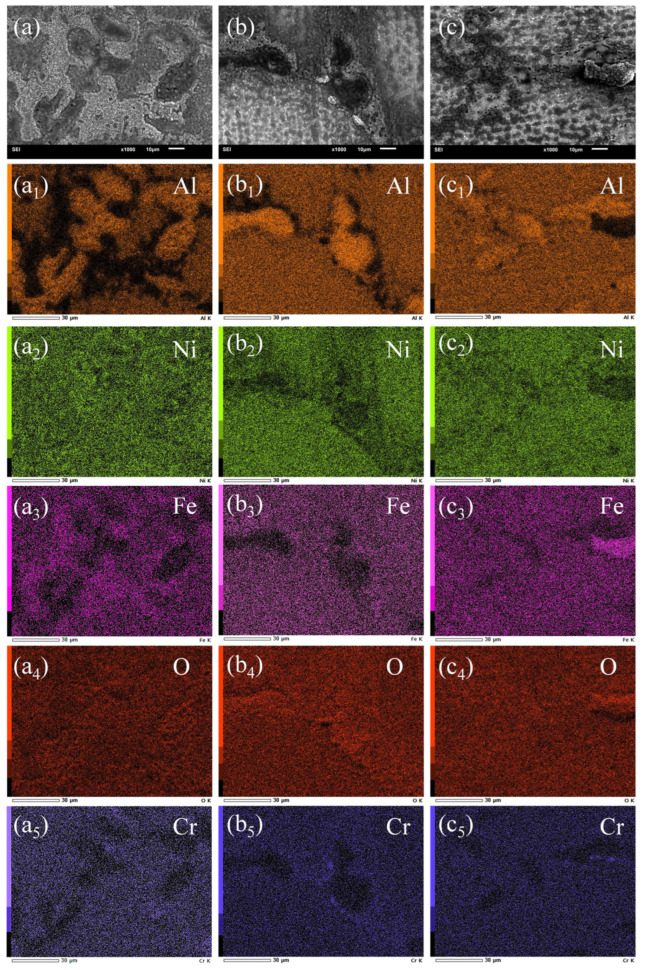
Elemental distribution of the original and pre-strained alloys after oxidation at 1100 °C for 30 min: (**a**,**a_1_**–**a_5_**) original alloy; (**b**,**b_1_**–**b_5_**) 5% pre-strained alloy; (**c**,**c_1_**–**c_5_**) 25% pre-strained alloy.

It should be noted that the oxidation products in different regions of the pre-strained specimens exhibit noticeable differences. The distribution of oxidation products after 30 min of oxidation in each region of the original and pre-strained alloys was analyzed using EDS mappings (Figure 9). In the β phase, aluminum elements tend to aggregate, while its absence is observed in the γ′-envelope near the β phase. Meanwhile, the absence of an aluminum element decreases with pre-strain increasing. Furthermore, Fe and Ni elements are enriched in the γ′-envelope region and the aggregation of Fe elements decreases with pre-strain increasing. The XRD and Raman detection results show the formation of NiFe_2_O_4_ in the γ′-envelope regions, while pre-strain appears to hinder the growth of NiFe_2_O_4_ and the spinel oxide cannot act as protective layer for the matrix [10]. In addition, the XRD and Raman results reveal that the oxidation products in the β phase consist of Al_2_O_3_ and NiO due to the aggregation of Al, Ni and O elements, shown in Figure 9a_1_. It is important to note that the distribution of Al elements is more even in the 25% pre-strained alloy compared to the 5% pre-strained alloy. The more uniform distribution of the Al element with increased pre-strain leads to more uniform formation of Al_2_O_3_. However, it is observed that the 25% pre-strained alloy generates more NiO in the β phase than the 5% pre-strained alloy (Figure 9b_2_,c_2_) and NiO has a weak protective effect on the matrix [46]. As described above, for the alloy with pre-strain of 5%, the pre-strain process does not only inhibit the formation of NiO and NiFe_2_O_4_ but also promotes the uniform formation of protective Al_2_O_3_, which positively contributes to its antioxidant properties.

### 4.3. Growth Behavior of Long-Term Cyclic Oxidation Products

**Figure 10:**
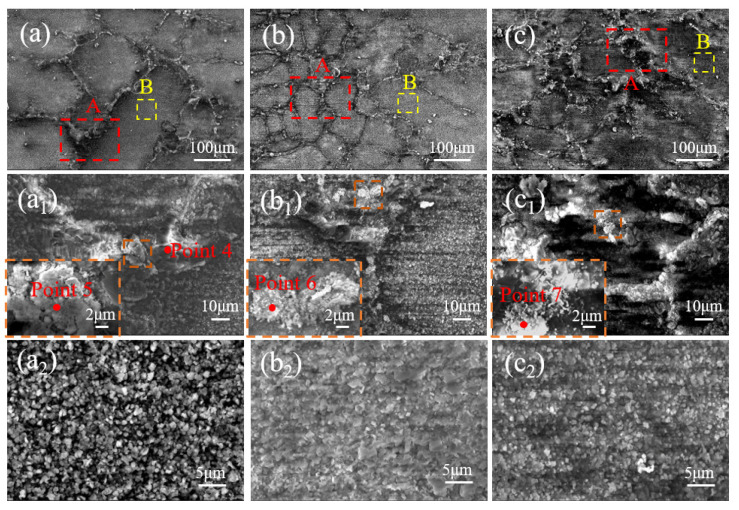
Oxidation morphologies of the original and pre-strained alloys after oxidation at 1100 °C for 200 h: (**a**) original alloy; (**a_1_**) original alloy_ Class A regions; (**a_2_**) original alloy_ Class B regions; (**b**) 5% pre-strained alloy; (**b_1_**) 5% pre-strained alloy_ Class A regions; (**b_2_**) 5% pre-strained alloy_ Class B regions; (**c**) 25% pre-strained alloy; (**c_1_**) 25% pre-strained alloy_ Class A regions; (**c_2_**) 25% pre-strained alloy_ Class B regions.

**Figure 11:**
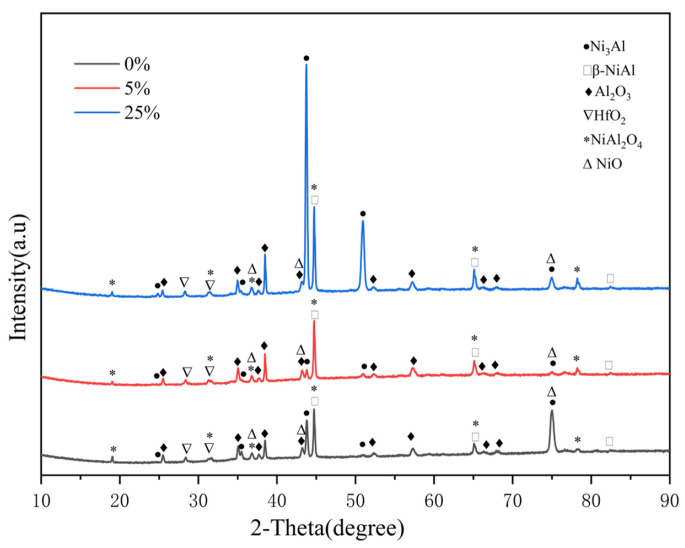
Phase composition of the original and pre-strained alloys after oxidation at 1100 °C for 200 h.

Figure 10 illustrates the surface morphologies of the original and pre-strained alloys after 200 h of cyclic oxidation at 1100 °C. The results show that the distribution of oxidation products becomes more uniform for the alloy with pre-strain of 5% compared to the original alloy (Figure 10b). Increasing the pre-strain to 25% results in a significant increase and aggregation of oxidation products (Figure 10c). To further analyze the oxidation products in different regions, the oxidized areas were classified into Class A and Class B (Figure 10a–c). It is clear that the Class A regions show denser oxidation morphology. Combining XRD (Figure 11) and EDS (Figure 10a_1_) detection results, it is determined that the primary oxidation product in the Class A regions is Al_2_O_3_. Additionally, flower-like oxides appear near Class A regions, as depicted in the enlarged Figure 10a_1_,b_1_,c_1_. EDS analysis reveals the presence of HfO_2_ in the flower-like oxides. As discussed in Section 3.2, HfO_2_ is predominantly located in the γ′-envelope region. The β phase adjacent to the γ′-envelope region possesses a higher Al content, which promotes the formation of Al_2_O_3_. It is suggested that Class A regions correspond to the β phase and γ′-envelope regions. Previous research has demonstrated that HfO_2_ is predominantly located at the interface, facilitating the rapid formation of Al_2_O_3_, anchoring the interface and enhancing the adhesion of oxide film [47,48,49]. Thus, it can be deduced that Class B regions correspond to the (γ + γ′) dendrite regions. Based on the XRD and EDS analysis, it is evident that the oxidation products in the (γ + γ′) dendrite region mainly consist of Al_2_O_3_, exhibiting a predominantly granular morphology (Figure 10a_2_). In the original alloy, the granular oxides are more dispersed. However, the morphologies of oxides transform from granular to a more compact scale-like structure in the 5% pre-strained alloy. When the pre-strain reaches 25%, the oxide changes to finely dispersed granular.

**Figure 12:**
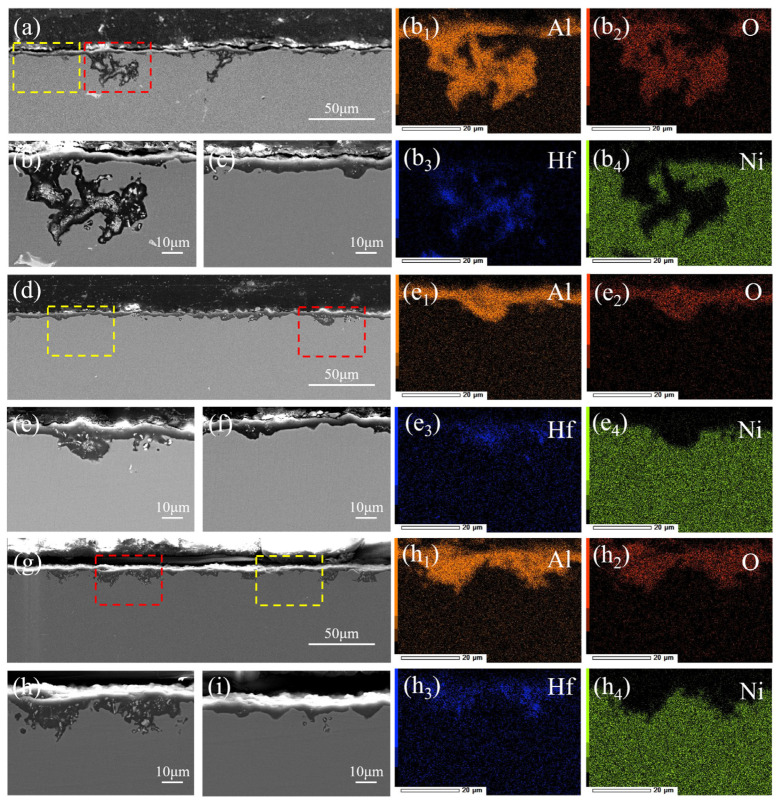
Cross-sectional morphologies of the original and pre-strained alloys after oxidation at 1100 °C for 200 h: (**a**,**b**,**c**) original alloy; (**b_1_–b_4_**) EDS mapping of figure b; (**d**,**e**,**f**) 5% pre-strained alloy; (**e_1_**–**e_4_**) EDS mapping of figure e; (**g**,**h**,**i**) 25% pre-strained alloy; (**h_1_**–**h_4_**) EDS mapping of figure h. The red boxes show the areas of deep oxidation. The yellow boxes show the areas of shallow oxidation.

Figure 12 illustrates the cross-sectional morphologies of the original and pre-strained alloys after 200 h of cyclic oxidation at 1100 °C. It is evident that after 200 h of cyclic oxidation, both the original and pre-strained alloys exhibit deep oxidation areas (Figure 12a,d,g). The oxidation depth for the 5% pre-strained alloy is significantly lower, showing more uniformly distributed oxidized deeper regions compared to the original alloy, while for the alloy with pre-strain of 25%, there is a notable increase in deep oxidation regions, despite a decrease in oxidation depth. To analyze the composition of the oxidation products in the deep oxidation regions, EDS mapping analysis was conducted. Combined with the XRD results (Figure 11), it is evident that the deep oxidation region is primarily composed of HfO_2_ which is surrounded by Al_2_O_3_ which corresponds to HfO_2_ near Al_2_O_3_ in Class A regions of the oxidized surface (Figure 10), suggesting that the oxidations in deep regions are primarily the result of HfO_2_ formed near the γ′-envelope. It has been proved that HfO_2_ enhances the adhesion of oxide films [47,48,49], while excessive oxide growth near HfO_2_ can increase the risk of spalling of oxidized products. Hence, it can be concluded that a well-dispersed HfO_2_ in the 5% pre-strained alloy is more beneficial for enhancing the cohesiveness of the oxide film.

### 4.4. Growth Mechanism of Oxidation Products

**Figure 13:**
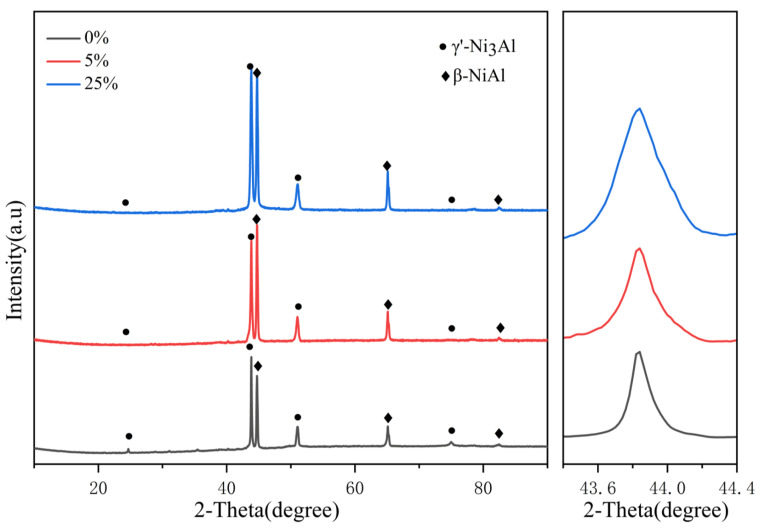
Phase composition of the original and pre-strained alloys.

Figure 13 shows the XRD detection results of the original and pre-strained alloys. It can be seen that the original and pre-strained alloys mainly consist of γ′ and β phases, meaning that the pre-strain process has little effect on the phase composition of the alloys. To investigate the impact of pre-strain on the defects of the alloys, the peaks on the (111) faces of γ′ phase were analyzed. It was observed that the peaks experiencing higher strain are broader compared to those with lower strain, as shown in the magnified view in Figure 13. The broadening of peaks is attributed to the presence of more defects, such as dislocations and stacking faults [29]. These defects act as sites for the nucleation of oxidation products during the oxidation process and significantly impact the antioxidant properties of the alloy [50].

**Figure 14:**
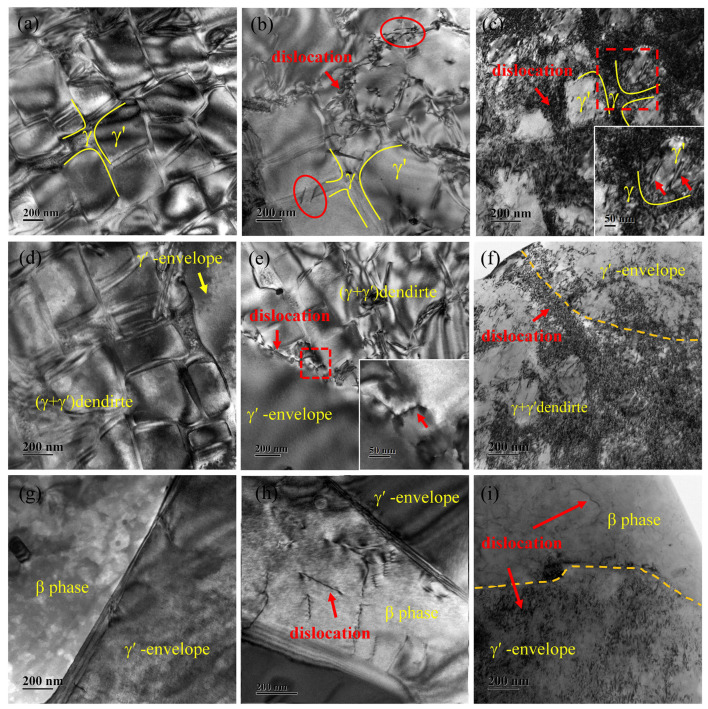
Effect of pre-strain on the dislocation of polycrystalline Ni_3_Al-based alloys: (**a**,**d**,**g**) original alloy; (**b**,**e**,**h**) 5% pre-strained alloy; (**c**,**f**,**i**) 25% pre-strained alloy. The red boxes show the enlarged area of the image.

To investigate the effect of pre-strain on the oxidation behavior of polycrystalline Ni_3_Al-based alloys, TEM tests were performed on the original and pre-strained alloys to assess their internal defects. Figure 14 shows the TEM images of each region for the original and pre-strained alloys. The results reveal that the dislocation quantity increases with the level of pre-strain. In the (γ + γ′) dendritic region, 5% pre-strain causes dislocations to primarily exist in γ channels, with difficulty passing through the γ′ phase (Figure 14a–c). However, there is a tendency for dislocations to climb up to the γ′ phase, as indicated by the red circles in Figure 14b. On the other hand, when pre-strain reaches 25%, dislocations primarily plug to γ channels (Figure 14c), with some regions exhibiting dislocations and cutting of the γ′ phase, as shown by the red arrows in Figure 14c. In the β phase and γ′-envelope regions, the number of dislocations increases with pre-strain. Meanwhile, the dislocation states differ due to structural variations between the two sides of the γ′-envelope. On the side near the (γ + γ′) dendrite, dislocations primarily locate in γ channels between the γ′-envelope and (γ + γ′) dendrite (Figure 14d–f). It is challenging for dislocations to cross the γ′-envelope at low strain (5%). When the pre-strain reaches 25%, a significant number of dislocations generate in the γ channel and γ′-envelope, similar to the region of the (γ + γ′) dendrite. On the side near the β phase, there are fewer dislocations at the lower pre-strain (5%), but dislocation entanglement appears when the pre-strain reaches 25%.

**Figure 15:**
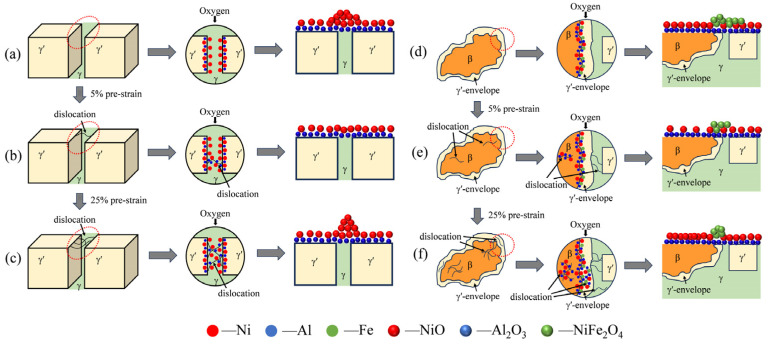
Schematic diagram of short-term oxidation behavior in each region of the original and pre-strained alloys: (**a**,**d**) original alloy; (**b**,**e**) 5% pre-strained alloy; (**c**,**f**) 25% pre-strained alloy. The red circles indicate areas of microstructural change.

To clearly describe the influence of pre-strain on the formation of oxide films, a schematic diagram for short-term oxidation was established (Figure 15). As a protective oxidation product, the formation of Al_2_O_3_ follows:

(1) $$\frac{4}{3}\mathrm{Al}+O_{2}=\frac{2}{3}{\mathrm{Al}}_{2}O_{3},K=\frac{1}{a_{\mathrm{Al}}^{\frac{4}{3}}\times a_{O_{2}}}$$

where a is the activity coefficient and K is the equilibrium constant. During the oxidation process in the (γ + γ′) dendritic region of the original alloy, the Gibbs free energy of the resulting Al_2_O_3_ is more negative than NiO [21], indicating that Al has a greater affinity for oxygen than NiO. However, the growth rate of Al_2_O_3_ is lower than NiO [37,51], and the stoichiometric amount of NiO is smaller than Al_2_O_3_ which means it will not be severely depleted at the reaction front like Al. Therefore, before the formation of a continuous oxide film of Al_2_O_3_, NiO will continuously expand on the surface. This phenomenon is referred to as “transient oxidation” [52]. Additionally, due to the higher energy at the γ/γ′ interface, metal cations and oxygen anions more easily enrich [37]. The faster growth rate of NiO leads to rapid aggregation and growth in the γ/γ′ interface region where continuous Al_2_O_3_ has not yet formed, resulting in the formation of oxidation bumps (Figure 15a). When a 5% pre-strain is applied to the alloy, the dislocations in γ-channels serve as nucleation sites for oxidation and play a role in connecting the γ′ phase (as indicated by the red circles in Figure 14b), facilitating the rapid precipitation of protective Al_2_O_3_ film. This inhibits the growth of NiO and promotes more uniform distribution (Figure 15b). However, when the pre-strain reaches 25%, the significant number of dislocations in γ channels promotes the formation of Al_2_O_3_, but the rapid growth of NiO is also enhanced by dislocations, leading to its quick nucleation and growth in γ channels before successive formation of the Al_2_O_3_ film, leading to the reappearance of an oxidized bumpy morphology (Figure 15c). Meanwhile, the different oxidation behaviors on both sides of the γ′-envelope are mainly due to the different dislocation states. On the side near the (γ + γ′) dendrite, the oxidation mechanism is similar to that in the (γ + γ′) dendrite region because of the similar composition of the γ′ phase. However, on the side near the β phase, a more distinct interface is formed due to structural differences, leading to the rapid formation of Al_2_O_3_, NiO and Fe_2_O_3_. As the oxidation process progresses, the Al content (Al activity) in the subsurface of the alloy decreases, which requires an increase in oxygen activity to maintain equilibrium, as stated by the law of mass action (Equation (1)) [10]. When the oxygen activity surpasses a thermodynamically critical threshold for engaging in reactions with other elements, the oxide is transformed into Ni-Fe spinel oxide through the subsequent reactions:

(2) $$2\mathrm{Ni}+O_{2}+2{\mathrm{Fe}}_{2}O_{3}=2\mathrm{Ni}{\mathrm{Fe}}_{2}O_{4}$$

(3) $$\mathrm{NiO}+{\mathrm{Fe}}_{2}O_{3}=\mathrm{Ni}{\mathrm{Fe}}_{2}O_{4}$$

Meanwhile, the rapid diffusion pathway at the interface intensifies the oxidation in the γ′-envelope region, resulting in significant oxidation prominence (Figure 15d). When pre-strain is applied to the alloy, extended growth of NiFe_2_O_4_ is impeded by the rapid formation of Al_2_O_3_ (Figure 15e). It should be noted that NiFe_2_O_4_ was not observed during the extended cyclic oxidation process, which is potentially due to the growth and exfoliation dynamics of the oxide film. Further investigations on this matter will be conducted in future experiments. Moreover, in the β phase, a higher content of Al results in the generation of more Al_2_O_3_. However, due to the presence of “transient oxidation”, NiO grows towards the center of the β phase at the interface between the β phase and γ′-envelope, as evidenced by the distribution of Ni elements in Figure 9a_2_. Rapid formation of Al_2_O_3_ after applying 5% pre-strain inhibits the growth of NiO in the β phase and gives rise to a dense oxide film. Nevertheless, when 25% pre-strain is applied to the alloy, a large number of dislocations appear in the β phase and the difference in growth rate between NiO and Al_2_O_3_ increases the stress on the oxidation products of the β phase which leads to the rupture of oxide film (Figure 4f) and exacerbates its antioxidant properties.

## 5. Conclusions

This work investigated the impact of pre-strain on the oxidation behavior of polycrystalline Ni_3_Al-based alloys at 1100 °C. Conclusions are drawn as follows:

(1) Pre-strain alters the internal morphology of the alloy. As pre-strain increases, the morphology of the γ′ phase in (γ + γ′) dendrites transforms from massive to striped and triangular, with dislocations primarily appearing in γ channels. It is challenging for dislocations to pass through γ′ phases at low strain (5%). When pre-strain reaches 25%, some regions exhibit dislocations climbing up the γ′ phase. The dimension of the β phase decreases and it also experiences tensile deformation perpendicular to the strain direction. The folded morphology of the γ′-envelope becomes sharper, and dislocations are plugged at the interface between the β phase and γ′-envelope. Pre-strain increases the dislocation density within each region.

(2) Pre-strain does not significantly impact the type of oxidation products in each region of the alloys. However, significant differences in oxidation products are observed in each phase region during the short-term oxidation process. In the (γ + γ′) dendrite and β phase regions, the primary oxidation products are Al_2_O_3_ and NiO, while in the γ′-envelope region, the oxidation products mainly consist of Al_2_O_3_, NiO and NiFe_2_O_4_.

(3) In conclusion, 5% pre-strain improves the antioxidant properties of the alloys. During the short-term oxidation process, the dislocations within the 5% pre-strained alloys primarily act as connectors, facilitating rapid formation of Al_2_O_3_. After long-term cyclic oxidation, the 5% pre-strained alloys exhibit lower mass gain, shallower oxidation depth and more compact oxide film structure compared to the original alloy. Conversely, when the pre-strain reaches 25%, the increased dislocations, coupled with varying growth rates of oxidation products, lead to the rupturing of the oxide film and non-uniform growth of these products. After long-term cyclic oxidation, the 25% pre-strained alloys show increased mass gain compared to the original alloy.

## Figures and Tables

**Table 1:** Chemical composition of polycrystalline Ni_3_Al alloy (wt. %).

C	Cr	Al	Ti	Hf	W	Mo	B	Fe	Si	Mn	Ni
0.06–0.2	7.4–8.2	7.6–8.5	0.6–1.2	0.3–0.9	1.5–2.5	3.5–5.5	<0.05	<2	<0.5	<0.5	Bal.

**Table 2:** Chemical composition of different sites in the alloy.

Site	Composition from EDS (at. %)					
	Al	Cr	Fe	Ni	O	Hf
1	3.90	4.89	4.04	14.56	55.95	16.66
2	15.17	3.10	4.79	28.01	48.93	0.01
3	26.26	3.39	5.05	15.04	50.25	0.01
4	28.38	0.5	0.54	1.39	69.17	0.02
5	26.71	0.31	0.39	4.74	64.23	3.62
6	24.71	0.29	1.59	3.73	64.94	4.73
7	24.94	0.23	1.36	3.00	66.43	4.04

## Data Availability

The data presented in this study are available on request from the corresponding author (due to privacy).
